# The association between local meteorological changes and exacerbation of acute wheezing in Kandy, Sri Lanka

**DOI:** 10.1080/16549716.2018.1482998

**Published:** 2018-06-18

**Authors:** N. D. B. Ehelepola, Kusalika Ariyaratne, Amithe Jayaratne

**Affiliations:** a Department of Medicine, The Teaching (General) Hospital–Kandy, Kandy, Sri Lanka; b Outpatient Department, Lanka Hydraulic Institute, Moratuwa, Sri Lanka

**Keywords:** Wheezing, Meteorological factors, COPD, Asthma, temperature, rainfall, visibility, humidity, Barometric pressure, Sri Lanka

## Abstract

**Background:** Severe wheezing is a common medical emergency. Past studies have demonstrated associations between exacerbation of wheezing and meteorological factors and atmospheric pollution. There are no past studies from Sri Lanka that analyzed correlation between daily multiple meteorological variables and exacerbation of wheezing.

**Objectives:** To determine the correlations between daily counts of patients nebulized at the Outpatient Department (OPD) of Teaching Hospital – Kandy (THK) and local meteorological variables, and to explore the utility of that information.

**Design:** We considered daily counts of patients nebulized at the OPD of THK as an indicator of exacerbations of wheezing in the population catered to by this hospital. We determined the correlations between daily counts of patients nebulized at OPD and the following meteorological variables for four years: daily rainfall, minimum temperature, maximum temperature, diurnal temperature range, difference between maximum temperature and the temperature at 1800 hours, daytime humidity, nighttime humidity, barometric pressure and visibility. We utilized wavelet time series method for data analysis.

**Results:** All nine meteorological parameters studied were correlated with the daily counts of patients nebulized with average lag periods ranging from 5 to 15 days. Peaks of daily rainfall, maximum temperature, diurnal temperature range, difference between maximum temperature and the temperature at 1800 hours and daytime humidity were followed by peaks of counts of patients nebulized (positive correlations). Troughs of minimum temperature, nighttime humidity, barometric pressure and visibility were followed by peaks of patients nebulized (negative correlations).

**Conclusions:** The THK shall expect more patients with acute wheezing after extremes of weather. Minimum temperature has been consistently correlated with the exacerbation of respiratory symptoms in the past studies in other countries as well. Hence, prescribing the inhalation of more drugs on unusually cold days (prophylactically) may help prevent acute exacerbation of wheezing in patients on treatment for asthma and COPD.

## Background

Acute exacerbations of asthma and chronic obstructive pulmonary diseases (COPD), and severe respiratory tract infections are among the commonest medical emergencies. Those patients are presented to health care institutions with wheezing and other symptoms. Both the prevalence and severity of respiratory allergic diseases including asthma, have increased in the recent past, worldwide [1,2]. Genetic and environmental factors contribute to the predisposition of asthma. This rise of asthma is attributed to changes in environmental factors [1,2]. Meteorological factors and air pollution are well known to be associated with wheezing [1–5]. Meteorological factors influence air pollutant concentration in the air and both of them influence aeroallergen levels in the atmosphere [4,5].

**Figure 4.**
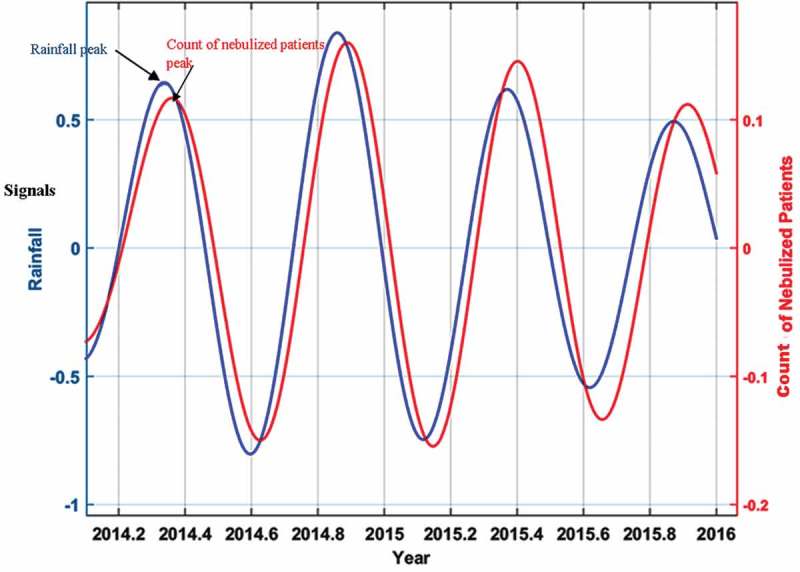
The wavelet filtered and reconstructed time series of rainfall versus the counts of nebulized patients. x axis – year, y axies: signals (primary y axis – rainfall, secondary y axis-count of the nebulized patients). The average time difference between the peaks of rainfall and counts of nebulized patients is the lag period.

In Sri Lanka, respiratory diseases were the second leading cause of hospitalization during 2003–2008 period and the third leading cause between 2009 and 2014 [6]. A considerable number of those patients were presented to outpatient departments (OPD) with wheezing.

South Asia, including Sri Lanka is undergoing urbanization, industrialization and the adoption of a Western lifestyle (e.g. the use of motor vehicles is on the rise). There is associated aggravation of atmospheric pollution. Those circumstances are reported to be associated with the rise of wheezing [1,2,7].

Studies done in other countries, mainly in the developed world show correlations between respiratory tract infections, asthma and COPD exacerbations and meteorological parameters of the locality [1–5,8]. But we cannot directly apply those findings to the Sri Lankan population as the genetic, socio-economic factors of our population, local weather and pollution patterns (atmospheric conditions) differ from those countries [1,4,9].

Despite the above background, only a few studies were done in Sri Lanka and in other parts of South Asia on the correlation between wheezing and atmospheric conditions [9,10]. Those Sri Lankan studies were also concerned only with air pollution (meteorological parameters were not considered). Global climate changes affect different countries to different extents [1,4]. To understand, predict and prepare for what is going to happen regarding wheezing in our society as a result of ongoing climate changes, we have to be aware of the existing association patterns between meteorological variables and wheezing. Considering all of the above, we undertook the present hospital OPD-based study.

There is no source of the daily atmospheric pollutants level data of Kandy or any other area of Sri Lanka for the duration of our study period. Hence we used a proxy, daily visibility level. Visibility is an indicator of the horizontal transparency of the atmosphere and is denoted as the horizontal distance at which a person should be able to see and identify an object [11]. The association between low visibility and respiratory morbidity and mortality was illustrated by studies done elsewhere [12–14].

The correlation between certain meteorological factors and exacerbation of wheezing is generally not linear and the correlation pattern does not remain constant throughout a time series (non-stationary). For example, if we consider rainfall, thunderstorms are known to exacerbate wheezing but drizzles are not [1], rain removes pollutants and pollen from air which reduces exacerbations of wheezing. Damp conditions after rain favor fungus growth in a few days and fungal spores are known to exacerbate wheezing [1,4]. We selected the wavelet time series analysis to study these correlations since it is a modern method more apt to analyze nonlinear and non-stationary time series [15].To the best of our knowledge this is the first time that technique was employed for a hospital-based study in South Asia of this nature.

## Study setting

THK (N7.286318, E80.631651,516 m) is the largest hospital in the Kandy district in the central highlands of Sri Lanka. Most of the patients visiting here with acute wheezing are residents of the most urban area of the Kandy district, living close to the hospital. The estimated population of the district in 2015 was 1,416,000. The vast majority of the buildings in Kandy do not have air conditioners or heaters, thus the indoor climate is also affected by the weather. Kandy city, where THK is situated, is in a basin surrounded by hills and mountains, resulting in the stagnation of polluted air. The Sri Lanka Department of Meteorology considers the data of the Katugastota weather station (approximately 5km North of THK) as representative of the Kandy district, therefore, data of Katugastota weather station was used in this study. The following metrological summary data give context to the conditions during the study period: The annual rainfall was 2108 mm. Average minimum temperature was 20.8°C, average maximum temperature was 29.3°C, average daytime relative humidity was 74.2%, average nighttime relative humidity was 92.5%,average visibility was 18.9 km and average barometric pressure was 958.1hectopascal.

## Objectives

Our broad objective was to identify the correlation between meteorological parameters and exacerbation of acute wheezing in the population catered by THK (a Sri Lankan community). We found that the daily counts of patients nebulized at the OPD of THK was the most practical indicator of daily exacerbation of acute wheezing in the population catered by THK for a retrospective study. This indicator had been used in the past in similar studies done in Colombo, Sri Lanka and in other countries [10,16,17]. Therefore, our specific objective was to study the correlation between daily counts of patients nebulized at the OPD of THK and daily local meteorological parameters.

Rainfall can promote or suppress exacerbation of acute wheezing, as explained above. Therefore we presumed in our time series, that after a peak of rainfall, either a peak or a trough of the count of patients nebulized can occur (either a positive or negative correlation is plausible). Minimum temperature and marked changes of temperature within a day were demonstrated to exacerbate wheezing in past studies [1,16,17], therefore we expected a peak of the count of patients nebulized after a trough of the minimum temperature (a negative correlation with minimum temperature). The difference between the daily maximum and minimum temperature is known as the diurnal temperature range (DTR). We expected positive correlations with DTR and the difference between maximum temperature and temperature at 1800 hours. (We expected a peak of the count of patients nebulized following a peak of DTR and the difference between maximum temperature and temperature at 1800 hours). High temperatures were shown to reduce lung functions in asthmatic children [18]. Thus we expected a peak of the count of patients nebulized after a peak of maximum temperature (a positive correlation). As stated above, low visibility is known to be analogous to poor air quality. Accordingly a peak of the number of patients nebulized was expected following a trough of visibility (a negative correlation with the visibility level). Both extremes of humidity were shown to exacerbate wheezing [1,19]. Hence a peak of the count of patients nebulized was expected to be plausible after a trough or a peak of relative humidity (either a positive or negative correlation with relative humidity). Past studies have demonstrated positive as well as negative correlations between barometric pressure and wheezing [20,21]. Thus we thought either a peak or a trough of the count of patients nebulized was plausible following a peak of barometric pressure.

## Methods

### Data

Ours is a retrospective descriptive study. We obtained the count (number) of patients (without any individually identifiable information) daily nebulized at the OPD of the THK from 1 January 2012 to December 31st of 2015 by going through hospital records. We purchased the daily data of rainfall, minimum and maximum temperature, daytime and nighttime humidity, barometric pressure and visibility data of the Katugastota weather station for the same period, from Sri Lanka department of meteorology.

### Analysis

Wavelet time series analysis (wavelet analysis) was employed to establish the association between the counts of patients nebulized daily and daily meteorological parameters. In wavelet analysis, an appropriate window is selected, it is shifted along the signal, and the spectrum is calculated for every position. This process is repeated several times with a slightly shorter or longer window for every new cycle. With the wavelet transform, the product will be a collection of time-frequency representations of the signal with diverse resolutions [15].

Wavelet transform can be utilized to study time series that contain non-stationary power at many different frequencies. We can establish both the dominant modes of variability and how these vary in time by decomposing a time series into a time-frequency space. Continuous wavelet transform (CWT) is a better way for feature extraction purposes, while Cross-wavelet transform (XWT) and wavelet coherence (WTC) can be used for observing relationships in time-frequency spaces between two time series. We can employ phase-angle statistics to gain confidence in causal relationships and to look for physical relationships between the time series [15].

CWT is a common tool for determining localized intermittent oscillations in a time series. It is preferable to examine two time series that may be interrelated in some way together. (To observe whether regions in time-frequency space with a large common power have a consistent phase relationship, and hence indicate causality between the two time series.)

We calculated Continuous Wavelet Transform (CWT) for each meteorological variable being studied. CWT expands the time series into time frequency space. It is a convolution with a wavelet which can be stretched and translated with resolutions in both frequency and time. The CWT decomposes the time series into time frequency space, enabling the identification of both the dominant modes of variability and how those modes vary with time.

A wavelet is a function with zero mean that is localized in both frequency and time.

In the present analysis, the Morlet wavelet was used,

Morlet wavelet: $\psi_{0}\left(\eta \right)=\pi^{-1/4}e^{i\omega_{0}\eta }e^{\frac{-1}{2}\eta^{2}}$

Where $\omega_{0}$ is dimensionless frequency and η is dimensionless time.

When using wavelets for feature extraction purposes the Morlet wavelet with $\omega_{0}$=6 is a good choice, since it provides a good balance between time and frequency localization [22].

The idea behind the CWT is to apply the wavelet as a bandpass filter to the time series. The wavelet is stretched in time by varying its scale, s, so that η= st, and normalizing it to have the unit energy.

The CWT of a time series, $X_{n}$, n = 1,2,…,N with uniform time step δt, is defined as the convolution of $X_{n}$ with the scaled and normalized wavelet [15,23].

$$W_{n}^{X}\left(s\right)=\sqrt{\frac{\delta t}{s}}\overset{N}{\underset{n^{\prime }=1}{\sum }}X_{n^{\prime }}\psi_{0}\left[\left(n^{\prime }-n\right)\frac{\delta t}{s}\right]$$

Wavelet power [15,23]:

$${\left|W_{n}^{X}\left(s\right)\right|}^{2}$$

The CWT has edge effects since the wavelet was not completely localized in time. Since a finite length time series is available, errors will occur at the beginning and end of the wavelet power spectrum, as the transform assumes the data is cyclic. Hence it is necessary to introduce Cone of Influence (COI) in which edge effects cannot be ignored. One solution is to pad the end of the time series with zeroes before the wavelet transform and then remove them afterwards. In this study the time series is padded with sufficient zeroes to bring the total length up to the next higher power of two. COI is the region of the wavelet spectrum in which edge effects become important. In the present analysis COI was taken as the area in which the wavelet power caused by a discontinuity at the edge has dropped to that of the value at the edge. The statistical significance is estimated against a red noise model. The thick black line is the 5% significance level using the red noise signal model and the thin black line indicates the cone of influence. Comparison of the CWT of the number of nebulized patients with weather parameters reveals clearly common features in the wavelet power. To verify the possibility of common power, we calculated the Cross wavelet Transform (XWT).

XWT finds regions in time frequency space where the time series show high common power.

The cross wavelet transform of two time series $X_{n}$ and $Y_{n}$ is defined as [23].

$$W^{XY}=W^{X}W^{Y*},$$

where * denotes complex conjugation.

Cross wavelet power [23]: $\left|W^{XY}\right|$

The vectors indicate the phase difference. A horizontal arrow pointing from left to right signifies in the phase and an arrow pointing vertically upward means the second series lags behind the first by 90°.

Based on XWT and XWT power variations, it is possible to have a causality relationship among the number of nebulized patients with weather parameters. In order to check the possibility of having causality effect, wavelet coherence was calculated.

Wavelet coherence is defined as the square of the cross spectrum normalized by the individual power spectra. This gives a quantity between 1 and 0, and measures the cross correlation between two time series as a function of frequency.

The Wavelet Coherence (WTC) finds regions in time frequency space where the two time series co-vary, but do not necessarily have high power. Coherence is a measure of the intensity of the covariance of the two series in time frequency space, unlike the cross wavelet power which is a measure of the common power. It was the aim to examine two time series together that was expected to be linked in some way. If there are regions in time frequency space with large common power, having a consistent phase relationship it suggests that causality between time series [23].

Wavelet coherence [23]:

$$R_{n}^{2}\left(s\right)=\frac{{\left|S\left(s^{-1}W_{n}^{XY}\left(s\right)\right)\right|}^{2}}{S\left(s^{-1}{\left|W_{n}^{X}\left(s\right)\right|}^{2}\right).S\left(s^{-1}{\left|W_{n}^{Y}\left(s\right)\right|}^{2}\right)}$$

Where S is a smoothing operator.

Notice that this definition closely resembles that of a traditional correlation coefficient, and it is useful to think of the wavelet coherence as a localized correlation coefficient in time frequency space.

For there to be a simple cause and effect relationship between the counts of nebulized patients and meteorological parameters, it is necessary that the oscillations are phase locked. That means they are required to have the same phase angle across the considered period. If they vary between in phase and anti-phase then it is a clue that they are probably not linked.

Examining the WTC and the phase arrows gives an indication that there could be a connection between the count of nebulized patients and weather parameters. In order to find the leading or lagging time the time series were reconstructed for the period which gave maximum power.

Since the wavelet transform is a bandpass filter with a known wavelet function, it is possible to reconstruct the original time series.

Reconstructed time series:

$$x_{n}=\frac{\delta j\delta t^{\frac{1}{2}}}{C_{\delta }\psi_{0}\left(0\right)}\overset{J}{\underset{j=0}{\sum }}R$$

Where, $\psi_{0}\left(0\right)$ removes the energy scaling, while $s_{j}^{\frac{1}{2}}$ converts the wavelet transform to an energy density. The factor $C_{\delta }$ is a constant for each wavelet function [24].

Considering the above equation and by summing a subset of the scales, it is possible to construct a wavelet filtered time series. In the present study, the period which gave the highest coherence among the count of nebulized patients and each of the meteorological parameter was identified. The wavelet filtered time series for this period were reconstructed and the average lagging time was estimated.

We used MATLAB R2013a software (MATLAB Corporation, USA) for the wavelet analysis.

## Results

**Figure 1.**
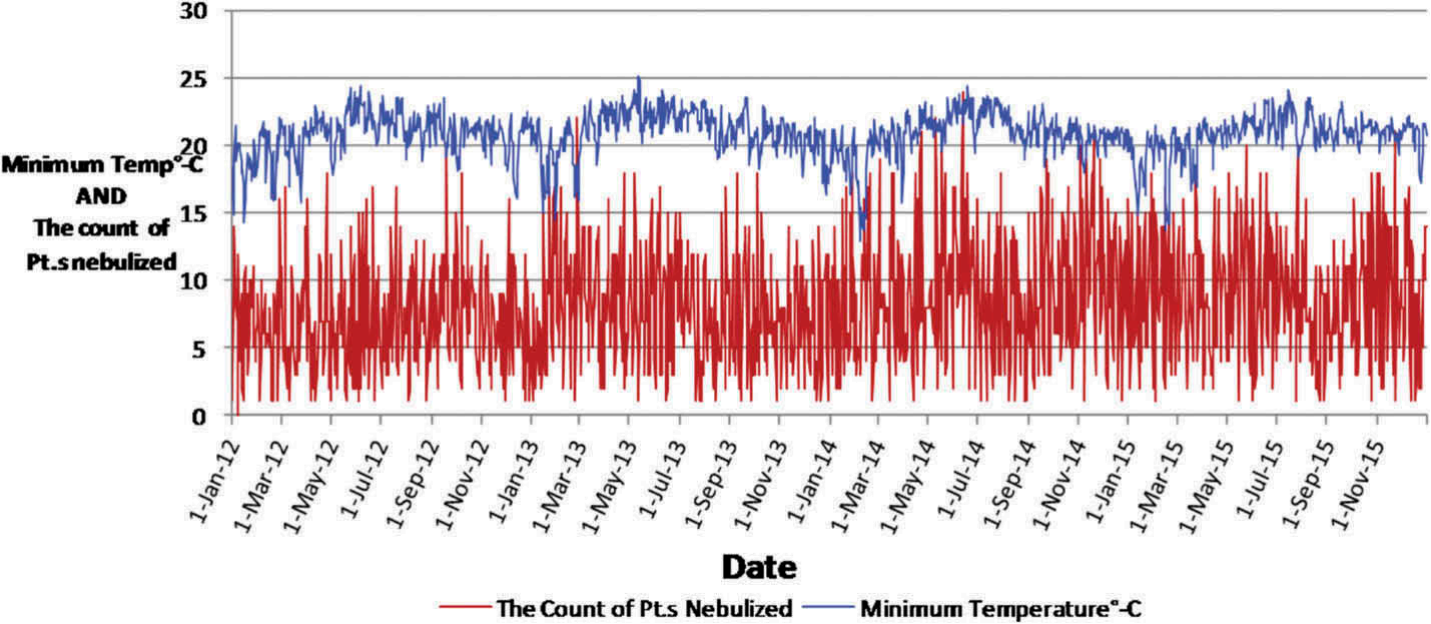
Changes of the minimum temperature (in °C) and the counts of patients nebulized at the OPD over the course of our period of study (2012–2015).x-axis: Date, y-axis: minimum temperature in degrees Celsius.

A total of 11,952 patients were nebulized at the OPD of the THK during our study period. Figure 1 is a time series graph depicting the daily changes of minimum temperature and the counts of patients nebulized during the period of our study, as an example.

**Figure 2.**
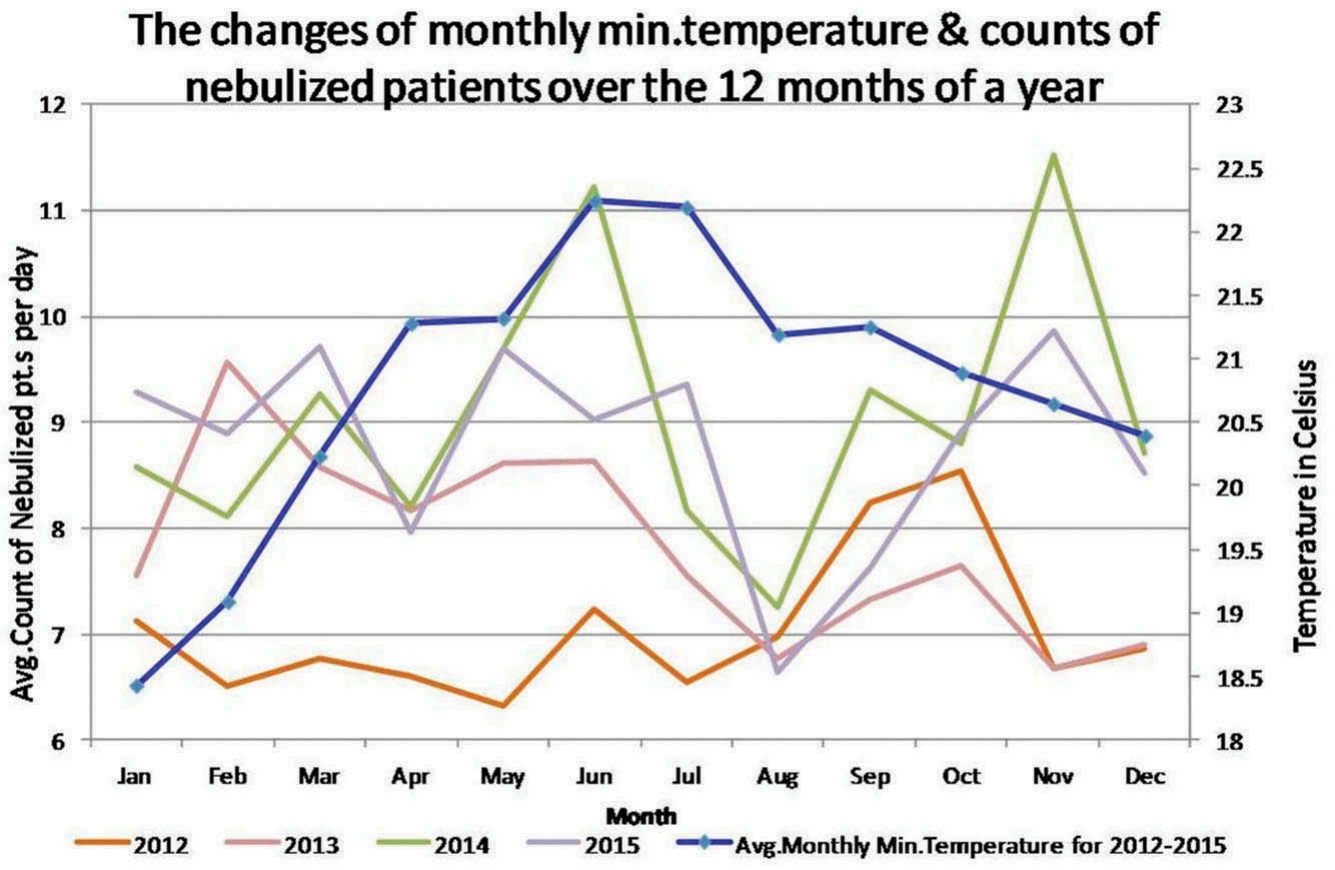
Changes of the averages of monthly minimum temperature (in °C) and the counts of patients nebulized at the OPD per day over the course of 12 months of year for our period of study (2012–2015). x-axis: Month, primary y-axis: Monthly average of the daily counts of patients nebulized, secondary y-axis: Average monthly minimum temperature in degrees of Celsius.

In Figure 1 there are large fluctuations of minimum temperature within a few days compared to the changes within a year and there are large fluctuations of counts of patients within a few days as well. The data appear noisy and the correlation between the two parameters is not obvious in Figure 1. This can be explained as several meteorological and other factors such as the introduction of a new strain of influenza virus to the Kandy population may influence the counts of patients nebulized. Our objective was to determine the correlation between daily data. Nevertheless four annual cycles of minimum temperature can be identified in Figure 1. Minimum temperature is low in the first three months of the year, peak during mid year and then gradually decline, during the course of all four years. We converted daily data into monthly data and calculated average monthly minimum temperature and monthly averages of daily counts of patients for 12 months and created Figure 2 to look for any visually obvious correlation between minimum temperature and the counts of patients nebulized.

Annual cycles of the minimum temperature is a well known regular occurrence. Thus we selected averages of the minimum temperature by month for the 2012–2015 for this graph and monthly patient counts for each year. We can see that there is a trough of patient counts in August in all four years and in April in three years. That indicates the occurrence of annual cycles of nebulized patient counts in this scale. From September to January the minimum temperature gradually declines and the patient counts usually rise. In January and February minimum temperature is low and the patient counts generally remain high compared to other months. In April the minimum temperature rises and patient counts drop. However, in May and June nebulized patients count rises despite of the high minimum temperature. We think this can be explained as the net effect of several factors that influence the count of patients. Nevertheless, the monthly scale results do not necessarily reflect the daily scale correlation.

**Figure 3.**
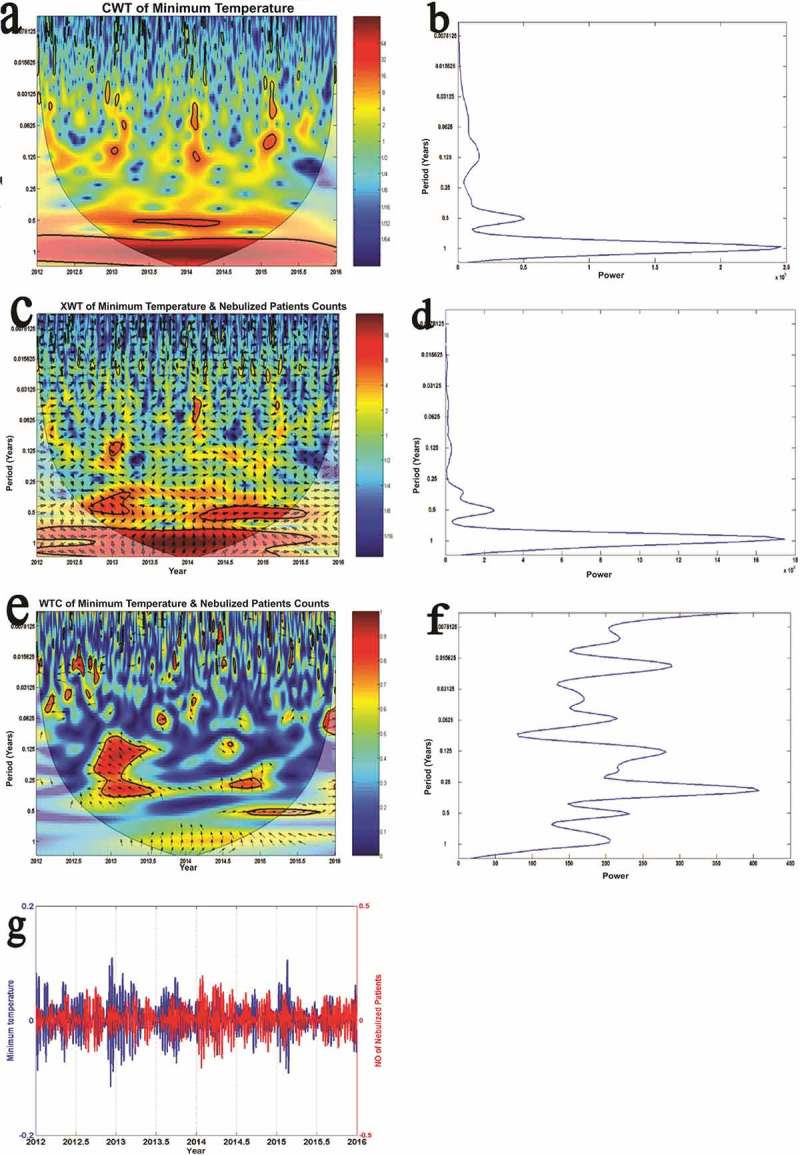
Results of wavelet analyses of daily minimum temperature versus the daily counts of the nebulized patients: (a) continuous wavelet transform (CWT) variations; (b) wavelet power of CWT; (c) crosswavelet transform (XWT) variations; (d) wavelet power of XWT; (e) wavelet coherence (WTC); (f) wavelet power of WTC; and (g) reconstructed time series for 2012–2016 period.

Figure 3 shows the results of wavelet analysis of daily minimum temperature versus daily counts of the nebulized patients as an example of our wavelet analysis results.

We would like to explain the Figure 3 further. Panel 3a shows the continuous wavelet transform of minimum temperature, which expands the time series into time frequency space, while panel 3b summarize the power for each period. Panel 3c shows the cross wavelet transform of minimum temperature with count of nebulized patients, where as panel 3d illustrates the power for each period. According to panel 3e and 3f, wavelet coherence is greatest between daily patient counts and daily minimum temperature during 2013, with quarterly cycle duration (period). The term ‘period’ in the vertical axis in panels 3a to 3f indicates the duration of a cycle (in years). There are color-coded panels on the right side of panels 3a, 3c and 3e. Those illustrate the magnitudes of CWT, XWT, and WTC, in which dark blue and dark red indicate the lowest and highest, respectively. The thin U shaped black lines in 3a,3c and 3e are the cone of influence which demarcates the area not influenced by edge effects. The thick black lines in panels 3a,3c and 3e are the 5% significance level using the red noise signal model. The vectors (arrows) indicate the phase difference in panels 3a,3c and 3e. A horizontal arrow pointing from left to right signifies in the phase and an arrow pointing vertically upward means the second series lags behind the first by 90°.

Panel 3g is the reconstructed time series for 2012–2016 period. As there were 133 cycles cramped in this panel the individual cycles are not clearly seen. The average time period between a trough of minimum temperature and subsequent peak in the count of patients nebulized in this time series denotes the lag period. Troughs of minimum temperature were followed by peaks of daily counts of patients nebulized (showing a negative correlation) after a lag period with an average five days. If peaks of a meteorological parameter (after a lag of a few days) were followed by peaks of the counts of patients nebulized that indicated a positive correlation. We use the reconstructed time series of rainfall versus the counts of nebulized patients (Figure 4) as a sample to further explain calculation of lag periods because that has few cycles and the reader can clearly see all of them.

For each cycle, in the reconstructed time series, the difference in time between the rainfall peak and counts of nebulized patients is calculated, and the average of this difference considering all the cycles in the reconstructed time series is estimated and included in Table 1. The same was repeated for all meteorological parameters.

**Table 1.** Summary of the results of wavelet analysis.

Daily Meteorological Parameter	The average lag periods in days and the range is within brackets	Correlation with daily count of patients nebulized
Rainfall (in millimeters)	11 *(8 – 15)*	A peak of rainfall is followed by a peak of the count of patients. (Positive Correlation)
Minimum temperature (in C°)	5 *(1 – 11)*	A trough of minimum temperature is followed by a peak of the count of patients. (Negative Correlation)
Maximum temperature (in C°)	5 *(1 – 10)*	A peak of maximum temperature is followed by a peak of the count of patients. (Positive Correlation)
Diurnal Temperature Range (in C°)	5 *(1 – 11)*	A peak of DTR is followed by a peak of the count of patients. (Positive Correlation)
Temperature difference between maximum temperature and temperature at 1800 hours (in C°)	15 *(1 – 24)*	A peak of temperature difference between maximum temperature and temperature at 1800 hours is followed by a peak of the count of patients. (Positive Correlation)
Daytime Relative Humidity (%)	4 *(1 – 8)*	A peak of daytime relative humidity is followed by a peak of the count of patients. (Positive Correlation)
Nighttime Relative Humidity (%)	9 *(1 – 16)*	A trough in nighttime relative humidity is followed by a peak of the count of patients. (Negative Correlation)
Barometric Pressure (in hpa)	10 *(1–20)*	A trough in barometric pressure is followed by a peak of the count of patients. (Negative Correlation)
Visibility (in km)	11 *(2–16)*	A trough in visibility is followed by a peak of the count of patients. (Negative Correlation)

The average lag periods vary from 5 to 15 days. In addition the range of lag periods are given within brackets.

## Discussion

Our results indicate that acute exacerbations of wheezing in Kandy are correlated with all nine of the local meteorological parameters we studied. The correlation patterns too were as we expected, except for nighttime humidity.

### Discussion of the biological basis of correlation patterns we derived for individual meteorological parameters and comparison with past studies showing similar results

We derived a positive correlation for rainfall. A similar recent study done in the USA. also demonstrated a positive correlation [25]. As described in the introduction, rain, especially thunderstorms, is known to exacerbate wheezing. Rain results in the swelling and rupture of atmospheric pollen grains, releasing pauci-micronic allergenic particles that are readily carried to the distal airways of lungs. Strong electric fields in the atmosphere that occur during thunderstorms may augment this process [1]. At the same time rain removes pollutant gases, particles and pollen from air which reduces exacerbations of wheezing [1]. Damp conditions after rain favor indoor and outdoor fungus growth in a few days and inhalation of fungal spores are known to exacerbate wheezing [1,4]. Considering the lag period of 11 days, a possible explanation of correlation we obtained is outdoor and indoor fungal growth after rain may be the dominant way rain affects wheezing in Kandy. However, further studies are necessary to come to a conclusion.

We have analyzed four temperature parameters and obtained a negative correlation for minimum temperature (a trough of minimum temperature is followed by a peak of the count of patients nebulized). Cold air can increase granulocyte and macrophage concentration in lower airways [1]. Provocation by cold air results in the degranulation of mast cells in the respiratory tract [26] releasing bioactive chemicals which can result in wheezing. Asthmatics and people with COPD are more prone to this phenomenon than the general population [1]. Asthmatics are more susceptible to nasal congestion when exposed to cold air as well, which leads to breathing via the mouth [1]. The nasal cavity warms up and filters the inhaled air. Oral breathing denies the warming and filtering of air. Exposure to cold air reduces the immunity to respiratory infections and decreases mucociliary clearance [1,27]. Low-ambient temperatures were demonstrated to result in similar respiratory illnesses in several countries in the recent past [1,3,8,27–29]. A drop of temperature even by 1°C was demonstrated to have led to acute exacerbations of COPD and that effect was pronounced with a larger (5°C) drop [8,29]. Nevertheless, there is no universal cut-off point below which we can define air as ‘cold’ or ‘warm’. It appears exposure to colder air than they are accustomed to exacerbate wheezing in people (the degree of temperature drop below the mean range for a given locality challenges the adaptive ability of the respiratory tracts of the residents of that locality) [1].

We obtained a positive correlation for maximum temperature. Maximum temperatures were revealed to be associated with higher levels of ground-level ozone [25]. Ground-level ozone is the main component of photochemical oxidants and the dominant contributor to oxidant levels in the atmospheres of many cities [2]. Inhaled ozone can tilt the oxidant-antioxidant balance towards a proinflammatory state in the respiratory systems of patients with asthma and COPD [1]. Ozone activates stress signaling pathways in epithelial cells and resident alveolar inflammatory cells of lungs with a resulting release of chemical mediators that precipitate wheezing [1]. Exposure to ozone results in the impairment of lung function, increased airway reactivity to nonspecific and specific bronchoconstrictor agents and thereby increases the risk of asthma and COPD exacerbations [2,25,30]. In one study, ambient temperature was negatively related to both morning and evening peak expiratory flow (PEF) and forced expiratory volume in the first second (FEV1) with 0–3 days lag [18]. Exposure to extreme heat was associated with increased risk of hospitalization for asthma in the USA, Australia and Europe [25,29,31]. However, their lag periods were shorter. Differences in patient factors or differences in the dominant mechanism of the action of high temperatures in Kandy may be plausible explanations. Further studies may give us a clearer picture.

We detected a positive correlation between DTR and the count of patients nebulized. DTR stands out among other meteorological parameters because the global average DTR is described as an index of global climate changes [32]. Studies done in Argentina and China have demonstrated that large DTRs were associated with increased risk of respiratory infections [33,34], and wheezing is one of the possible symptoms of respiratory infections. Studies from China and Greece indicate large DTRs may set off childhood asthma [35,36] and most interestingly a study from Hong Kong showed large DTRs independent from mean and minimum temperature and atmospheric pollutant levels increase acute asthma with 0–4 days lag [37]. Large DTRs were shown to be a risk of COPD mortality as well [38]. Our results generally agree with all those findings. Nevertheless the exact biological mechanisms by which exposure to large DTRs increases asthma risk is not yet understood [35]. The DTR becomes large when temperatures move to extremes either way within a day. Therefore, the biological mechanisms we explained before regarding minimum and maximum temperatures may play a role here as well. It is interesting to note with climate changes, the DTR can be inclined to reduce worldwide, with minimum temperatures rising faster than maximum temperatures [32]. The maximum temperature of the Katugastota weather station has risen faster than its minimum temperature in the recent past in contrast to the global trend even though both have risen [39].

The relationship between local short-term fluctuations of ambient temperature within a day and wheezing in the community was very rarely studied [40]. We have obtained a positive correlation with the temperature difference between maximum temperature and temperature at 1800 hours. Impairment of lung functions following cold, dry air challenges is a known phenomenon and even proposed as a diagnostic test for asthma in children [41]. Nevertheless, ambient temperatures do not change sharply in tropical countries (Sri Lanka) like in that test. The biological mechanisms we discussed in relation to minimum temperature may be applicable in this situation as well. One Japanese study demonstrated that larger changes of temperature in either direction were related to a higher risk of acute asthma attacks. Ambient temperature *per se* was not associated with acute asthma in that study [40]. A study from China showed large temperature drops were associated with a significant risk of respiratory infections [17]. The results of those studies are in agreement with the correlation we obtained for the difference between maximum temperature and temperature of 1800 hours analysis. In Sri Lanka the range of hourly temperature within a day is usually greater than the range of monthly mean temperatures within a year. Our results show the influence of daily variation of ambient conditions on the health of the vulnerable Kandy population.

We obtained a positive correlation with daytime relative humidity (a peak of relative humidity is followed by a peak of the count of patients) and a negative correlation (nighttime relative humidity (a trough of relative humidity is followed by a peak of the count of patients). The humidity of the air we breathe can affect the respiratory system directly (e.g. action of cilia) or indirectly by creating favorable conditions for the transmission of viral and bacterial infections of the respiratory system, effects on outdoor and indoor fungal growth and indoor dust mite growth, off-gassing of formaldehyde from indoor building materials, the rate of removal of sulfur and nitrogen dioxides from air by formation of acids and salts, the rate of formation of ground level ozone, swelling and rupture of pollen grains in the air and the formation of organic aerosol particles [1,19,42]. Inhalation of those pathogens, chemicals, body parts and excreta of dust mites and fungal spores can precipitate wheezing [1,19,42].To minimize those adverse health effects caused by humidity, maintaining indoor relative humidity levels in between 40% and 60% is recommended [42]. Most buildings of the Kandy area have no climate control and during the day time most people keep windows open hence the outdoor humidity has a greater influence on indoor humidity. During our study period the average daytime relative humidity was 74.2%, and average nighttime relative humidity was 92.5%, indicating the majority of the Kandy population was exposed to air with unhealthy levels of high relative humidity. A positive correlation between humidity and exacerbations of asthma and respiratory infections were obtained in some past studies [3,18,43].They all are in agreement with our results for daytime humidity. Most residents of Kandy stay at homes (in bed) during nights with closed windows and doors. The difference in the micro-environment most people get exposed to during nights and the differences of how those environments get influenced by humidity may be a reason for the different correlation pattern derived.

We could not find literature on the biological mechanisms of barometric pressure changes influencing exacerbations of wheezing. In one interesting study, moving to a site with high barometric pressure has improved arterial oxygenation in COPD patients without changing the spirometry results [44]. Research done in Japan and Britain have demonstrated negative correlation with acute exacerbations of asthma and barometric pressure which agree with our results [21,45].

We have observed a negative correlation with visibility, meaning that days with lower visibility had subsequent increases in counts of patients nebulized with a lag period of 11 days. Low visibility, especially in urban areas like Kandy, is known to correspond with poor air quality [12,13,46]. Air pollutant particulate matter, especially those with diameters <2.5μm, are known as the key factor responsible for impairment of atmospheric visibility. Pollutant gases like nitrogen dioxide (NO_2_) directly contribute; NO_2_ and sulfur dioxide indirectly contribute via particulate matter formation as well. Ground level ozone derived from reactions involving NO_2_ also contribute to poor visibility [2,45]. Inhalation of all of them can exacerbate wheezing in susceptible people [1]. Studies from China and Hong Kong showed reduced visibility as significantly correlated with mortality due to respiratory diseases and other causes [13,47]. A low visibility episode was associated with poor air quality and increased asthma and respiratory diseases in Brunei [14]. The results of those studies concur with our results. The mean visibility for our study period was 18.9 km indicating that generally the quality of air in Kandy is good [48]. This agrees with a study of pollutant levels of Kandy atmosphere [49]. However, Katugastota weather station is in the outskirts of the city area and not in close proximity to a major road. In the downtown area of Kandy city (surrounded by hills and mountains) especially closer to roads with high motor vehicle traffic and major bus terminals, there are localities with poor air quality [49,50]. Residing in the proximity of roads with high motor vehicle traffic was demonstrated to increase exacerbations of asthma [1]. According to the World Health Organization’s (WHO) global urban ambient air quality database-2016, 98% cities with populations>100,000 in low-and middle-income nations (like Kandy city) have ambient air that do not meet WHO standards [51]. It indicates the necessity of sustained air pollutant levels being monitored in downtown areas of Kandy city as well as in the peripheral areas. In the absence of the daily monitoring of key pollutant levels, then at least visibility levels (using a nephometer) need to be monitored regularly.

There are other studies on the correlation between meteorological factors and respiratory symptoms with results that differ from our results as well [1,8,19]. It is well known that all these meteorological parameters are interrelated.

## The implications/utility of the findings of this study

Our results show that the OPD staff of the THK shall expect more patients with acute wheezing following extremes of weather as per Table1. We believe these findings can be applicable to other hospitals in Kandy and may even to hospitals in other areas with a similar climate. Nevertheless the utility of our results are limited because we cannot give threshold levels beyond which the meteorological parameters are to be considered extreme. Further studies are needed to verify the applicability of our results to other localities and to enhance the utility of our results. Children and the elderly are more susceptible to respiratory symptoms due to atmospheric variable changes [1,2,52].The majority of past studies globally on this topic deal with pediatric populations. Sri Lanka has only two dedicated children’s hospitals and there are more than 600 hospitals with OPDs plus indoor care facilities catering to patients of all age groups, including the THK [6]. Hence we think our results are more relevant to the Sri Lankan set-up.

Minimum ambient temperatures were demonstrated to be associated with wheezing and similar respiratory illnesses in our study and several countries in the recent past [1,3,8,27–29]. According to the World Allergy Organization, cold air exacerbating asthma is consistent, unlike the correlation patterns between asthma and other meteorological parameters reported around the world [1]. Considering those results and ours, we think there is a place for recommending to colleagues in the Kandy area to ask their asthma and COPD patients to increase their inhaled drug doses (prophylactically) on unusually cold days. This recommendation may be applicable to other areas of Sri Lanka and elsewhere. We would prefer to do a pilot study first to confirm the efficacy of that recommendation. The maximum temperature has risen in Kandy and worldwide in the recent past due to ongoing climate changes [39]. That favors the precipitation of more acute attacks of wheezing. DTR is generally declining worldwide due to climate changes [32] and that is unfavorable for the precipitation of more acute attacks of wheezing. However, the final outcome of the effects of climate changes on wheezing in Kandy and elsewhere will be decided by the complex interplay of those two and several other factors [4].

Studies done in other countries have demonstrated that poor visibility is associated with poor air quality therefore with higher respiratory symptoms [13,14,47]. The mean visibility for our study period was18.9 km, indicating that generally the quality of air in the Kandy district is good [48]. This agrees with a study of pollutant levels of the Kandy atmosphere [49].

Developing nations, including Sri Lanka, are severely affected by air pollution [51]. However, there is not a single source of the daily air pollutant levels in Sri Lanka at present (2017). The generation of such data is a costly procedure, although it is very important. If visibility is proven to be a reliable proxy of air quality by further studies as well, that can be utilized to study the health hazards (like exacerbations of wheezing) of air pollution as we did among the vast and neglected populations of Sri Lanka and other developing nations.

Meteorological and other factors that influence exacerbations of wheezing vary from locality to locality as we described in the introduction. We believe more studies like this will help doctors be aware of the weather-wheezing correlation patterns of their locality and use that information to improve patient care. The methodology we adopted is a relatively new and novel one for studies of this nature. We hope the knowledge of this methodology would be useful to colleagues elsewhere.

## Limitations

The decision to nebulize a patient with acute wheezing at the OPD itself or send them to an appropriate ward for that purpose was a clinical decision taken by the individual doctor who attended the patient. There are individual variations in clinical decisions and which could have influenced our data set. There are no records of the permanent residence of our patients. Some may be not permanent residents of Kandy. There are local variations of weather [15] and air pollutant levels [49,50] within the Kandy district within short distances due to hill terrain and other factors. We only considered the data of Katugastota weather station. However, it is the only weather station in the Kandy district that daily records all the meteorological parameters we studied. Other important factors that could have exacerbated wheezing, like indoor air pollution, and smoking were not assessed in this study.

## Conclusions

Changes of several local meteorological parameters affect acute exacerbations of wheezing among the Kandy population. Hence the OPD staff shall expect more patients with acute wheezing after extremes of those meteorological parameters (as per Table1). We shall expect changes in patterns of exacerbation of wheezing in Kandy with ongoing climate changes. Minimum temperature correlated with exacerbation of respiratory symptoms in the present study and consistently in the past studies by other countries as well. Hence, taking a higher dose of inhaled drugs or inhaling more frequently during unusually cold days may help to control acute exacerbations of wheezing in patients with asthma and COPD.

## Paper context

We have studied the correlations between daily counts of patients nebulized at the outpatient department of a large Sri Lankan hospital (an indicator of acute wheezing attacks in that community) and 9 local meteorological variables for 4 years, for the first time. They were correlated with 5–15 day average lag periods. We compare our results with that of related studies, discuss relevant biological mechanisms and illustrate the utility of our findings.
